# Supplementary material for: Heat adaptation of phage T7 under an extended genetic code
Source: Virus Evol. 2021 Dec 1;7(2):veab100. doi: 10.1093/ve/veab100 (PMC8923235; doi:10.1093/ve/veab100)
Supplement: veab100_Supp [file veab100_supp.zip › Sequencing_Analysis.zip!/Sequencing_Analysis/A2/evidence/SNP_5.html]

BRESEQ :: Evidence


| Predicted mutation | | | | | | |
| --- | --- | --- | --- | --- | --- | --- |
| evidence | seq id | position | mutation | annotation | gene | description |
| RA | NC\_001604.1 | 20,544 | G→A | intergenic (–/–) | – / – | –/– |

| Read alignment evidence... | | | | | | | | | | | |
| --- | --- | --- | --- | --- | --- | --- | --- | --- | --- | --- | --- |
|  | seq id | position | | ref | new | freq | score (cons/poly) | reads | annotation | genes | product |
| \* | NC\_001604.1 | 20,544 | 0 | G | A | 99.8% | 1089.7 / ‑4.4 | 518 | intergenic (–/–) | *–/–* | –/– |
| Reads supporting (aligned to +/- strand):  **ref** base G (0/1);  **new** base A (242/275);  **total** (242/276) | | | | | | | | | | | |
| Fisher's exact test for biased strand distribution *p*-value = 1.00e+00 | | | | | | | | | | | |
| Kolmogorov-Smirnov test that lower quality scores support variant *p*-value = 9.60e-01 | | | | | | | | | | | |

Only 100 of 519 total aligned reads displayed.  

`GAGAAACGAACAGGACTTGCGGAGGATGGCGCAAAGTCTGTCTATGAGCGTTTAAAGAACGACCGTGCTCCCTATGAGACACGCGCTCAGAATTGCGCTCAATATACCATCCCATCATTGTTCCCTAAGGACTCCGATAACGCCTCTACAGATTATCAAACTCCGTGGCAAGCCGTGGGCGCTCGTGGTCTGAACAATCTAGCCTCTAAGCTCATGCTGGCTCTATTCCCTATGCAGACTTGGATGCGACTTACTATATCTGAATATGAAGCAAAGCAGTTACTGAGCGACCCCGATGGACTCGCTAAGGTCGATGAGGGCCTCTCGATGGTAGAGCGTATCATCATGAACTACATTGAGTCTAACAGTTACCGCGTGACTCTCTTTGAGGCTCTCAAACAGTTAGTCGTAGCTGGTAACGTCCTGCTGTACCTACCGGAACCGGAAGGGTCAAACTATAATCCCATGAAGCTGTACCGATTGTCTTCTTATGTGGTCCAACGAGACGCATTCGGCAACGTTCTGCAAATGGTGACTCGTGACCAGATAGCTTTTGGTGCTCTCCCTGAGGACATCCGTAAGGCTGTAGAAGGT  >  NC_001604.1/20246‑20839`  

                                                                                                                                                                                                                                                                                                          `|`                                                                                                                                                                                                                                                                                                         
      `cGAATAGGACTTGCGGAGGATGGCGCAAAGTCTGTCTATGAGCGTTTAAAGAACGACCGTGATCCCTATGAGACACGCGCTCAGAATTGCGCTCAATATACCATCCCATCATTGTTCCCTAAGGACTCCGATAACGCCTCTACAGATTATCAAACTCCGTGGCAAGCCGTGGGCGCTCGTGGTCTGAACAATCTAGCCTCTAAGCTCATGCTGGCTCTATTCCCTATGCAGACTTGGATGCGACTTACTATATCTGAATATGAAGCACAGCAGTTACTGAGCTACCCCGATGAAATCGcca                                                                                                                                                                                                                                                                                                 >  1:819047/1‑299 (MQ=255)`  
      `cGAACAGGACTTGCGGAGGATGGCGCAAAGTCTGTCTATGAGCGTTTAAAGAACGACCGTGCTCCCTATGAGACACGCGCTCAGAATTGCGCTAAATATACCATCCCATCATTGTTCCCTAAGGACTCCGATAACGCCTCTACAGATTATCAAACTCCGTGGCAAGCCGTGGGCGCTCGTGGTCTGAACAATCTAGCCTCTAAGCTCATGCTGGCTCTATTCCCTATGCAGACTTGGATGCGACTTACTATATCTGAATATGAAGCAAAGCAGTTACTGAGCGACCCCGATGAACTCGCTa                                                                                                                                                                                                                                                                                                 <  1:124855/301‑1 (MQ=255)`  
                `ctcggagatgtgtataagagacagtctATGAGCGTTTAAAGAACGACCGTGCTCCCTATGAGACACGCGCTCAGAATTGCGCTCAATATACCATCCCATCATTGTTCCCTAAGGACTCCGATAACGCCGCTACAGATTATCAAACTCCGTGGCAAGCCGTGGGCTCTCGTGGTCTGAACAATCTAGCCTCTAAGCTCATGCTGGCTCTATTCCCTATGCAGACTTGGATGCGACTTACTATATCTGAATATGAAGCAAAGCAGTTACTGAGCGACCCCGATGAACTCGCTAAGGTCAATGa                                                                                                                                                                                                                                                                                       <  1:754913/278‑1 (MQ=255)`  
                 `tGCGGAGGAAGGCGCAAAGGCTGTTAATCAGCGGTTAAAGAACGACCGTTCTCACTATGAGTCACGCGCTCAGAATTGCCCTAAATATACAAACCCATCAGTGTTCCCGAAGGACTCCGATAACGCCTCTACAGATGATCAAACTCCGTGGCAAGCCGTGGGCGCTCGTGGTCGGAAAGATCTAGTCTCTAAGCTCATGCTGGCTCTATTCCCTATGCAGACTTGGATGCGACTTACTATATCTGCATATGAAGCAAAGCAGTTACTGAGCGACCCCGATGAACTCGCTAAGGTCGATGAg                                                                                                                                                                                                                                                                                      <  1:80309/301‑1 (MQ=255)`  
                       `ggaTGGCGCAAAGTCTGTCTATGAGCGTTTAAAGAACGACCGTGCTCCCTATGAGACACGCGCTCAGAATTGCGCTCAATATACCATCCCATCATTGTTCCCTAAGGACTCCGATAACGCCTCTACAGATTATCAAACTCCGTGGCAAGCCGTGGGCGCTCGTGGTATGAACAATCTAGCCTCTAAGCTCATGCTGGCTCTATTCCATATGCAGACTTGGATGCGACTTACTATATCTGAATATGAAGCAAAGCAGTTACTGAGCGACCCCGATGAACTCGCTaactgtctcttatacaca                                                                                                                                                                                                                                                                                >  1:275143/1‑285 (MQ=255)`  
                             `cgcAAAGTCTGTCTATGAGCGTTTAAAGAACGACCGTGCTCCCTATGAGACACGCGCTCAGAATTGCGCTCAATATACCATCCCATCATTGTTCCCTAAGGACTCCGATAACGCCTCTACAGATTATCAAACTCCGTGGCAAGCCGTGGGCGCTCGTGGTCTGAACAATCTAGCCTCTAAGCTCATGCTGGCTCTATTCCCTATGCAGACTTGGATGCGACTTACTATAACTGAATATGAAGCAAAGCAGTTACTGAGCAACCACGATGAACTCGCTAAGGTCGATGAGGGCCTCTCGaag                                                                                                                                                                                                                                                                          >  1:214136/1‑299 (MQ=255)`  
                               `cAAAGTCTGTCTATGAGCGTTTAACGAACGACCGTGCTCCCTATGAGCCACGCGCTCAGAATTGCGCTCAATATCCCATCCCATCATTGTTCCCTAAGGACTCCGATAACGCCTCTACAGATTATCCAACTCTGTGGCAAGCTGTGGGCTCTCGTGGTCTTAACAATCTATCCTCTAAGCTCATGCTGGCTCTATTCCCTATGCAGACTTGGATGCGACTTACTATATCTGAATATGAAGCAAAGCAGTTACTGAGCGACCCCGATGAACTCGCTAAGGTCGATGAGGGCCTCTCGATGGt                                                                                                                                                                                                                                                                        <  1:471756/301‑1 (MQ=255)`  
                               `atgtgtataagagacaGCGTTTAAAGAACGACCGTGCTCCCTATGAGACACGCGCTCAGAATTGCGCTCAATATACCATCCCATCATTGTTCCCTAAGGACTCCGATAACGCCTCTACAGATTATCAAACTCCGTGGCAAGCCGTGGGCGCTCGTGGTCTGAACAATCTAGCCTCTAAGCTCATGCTGGCTCTATTCCCTATGCAGACTTGGATGCGACTTACTATATCTGAATATGAAGCAAAGCAGTTACTGAGCGACCCCGATGAACTCGCTAAGGTCGATGAGGGCCTCTCGATGGt                                                                                                                                                                                                                                                                        <  1:393498/286‑1 (MQ=255)`  
                                   `gtctgtctATGAGCGTTTAAAGAACGACCGTGCTCCCTATGAGACACGCGCTCAGAATTGCGCTCAATATACCATCCCATCATTGTTCCCTAAGGACTCCGATAACGCCTCTACAGATTATCAAACTCCGTGGCAAGCCGTGGGCGCTCGTGGTCTGAACAATCTAGCCTCTAAGCTCATGCTGGCTCTATTCCCTATGCAGACTTGGATGCGACTTACTATATCTGAATATGAAGCAAAGCAGTTACTGAGCGACCCCGATGAACTCGCTAAGGTctgtctcttatacacatctccgagc                                                                                                                                                                                                                                                                    >  1:433479/1‑277 (MQ=255)`  
                                   `gtctgtctATGAGCGTTTAAAGAACGACCGTGCTCCCTATGAGACACGCGCTCAGAATTGCGCTCAATATACCATCCCATCATTGTTCCCTAAGGACTACGATAACGCCTCTACAGATTATCAAACTCCGTGGCAAGCCGTGGGCGCTCGTGGTCTGAACAATCTAGCCTCTAAGCTCATGCTGGCTCTATTCCCTATGCAGACTTGGATGCGACTTACTATATCTGAATATGAAGCAAAGCAGTTACTGAGCGACCCCGATGAACTCGCTAAGGTCGATGAGGGccctgtctcttataca                                                                                                                                                                                                                                                                    >  1:437754/1‑287 (MQ=255)`  
                                   `gtctgtctATGAGCGTTTAAAGAACGACCGTGCTCCCTATGAGACACGCACTCAGAATTGCGCTCAATATACCATCCCATCATTGTTCCCGAAGGACTCCGATAACGCCTCTACAGATTATCAAACTCCGTGGCAAGCCGTGGGCGCTCGTGGTCTGAACAATCTAGCCTATAAGCTCATGCTGGCTCTATTCCCTATGCAGACTTGGATGCGACTTACTATATCTGAATATGAAGCAAAGCAGTTACTGAGCGACCACGATGAACTCGCTAAGGTCGATGAGGGCCTCTCGATGGTagag                                                                                                                                                                                                                                                                    <  1:204605/301‑1 (MQ=255)`  
                                       `gtctATTAGCGTTTAAAGAACGACCGTGCTCCCTATGAGACACGCGCTCAGAATTGCGCTCAATATACCATCCCATCATTGTTCCCTAAGGACTCCGATAACGCCTCTACAGATTATCAAACTCCGTGGCAAGCCGTTGGCGCTCGTGGTCTGAACAATCTAGCCTCTAAGCTCATGCTGGCTCTATTCCCTATGCAGACTTGGATGCGACTTACTATATCTGAATATGAAGCAAAGCAGTTACTGAGCGACCCCGATGAACTCGCTAAGGTCGATGAGTGCCTCTCGATGGTagctgtct                                                                                                                                                                                                                                                                >  1:503384/1‑295 (MQ=255)`  
                                       `gtctATGAGCGTTTAAAGAACGACCGTGCTCCCTATGAGACACGCGCTCAGAATTGCGCTCAATATACCATCCCATCATTGTTCCCTAAGGACTCCGATAACGCCTCTACATATTATCAAACTCCGTGGCAAGCCGTGGGCGCTCGTGGTCTGAACAATCTAGCCTCTAAGCTCATGCTGGCTCTATTCCCTATGCAGACTTGGATGCGACTTACTATATCTGAATATGAAGCAAAGCAGTTACTGAGCGACCCCGATGAACTCGCTAAGGTCGATGAGGGCCTCTCGATGGTAGATCGTa                                                                                                                                                                                                                                                                >  1:346008/1‑301 (MQ=255)`  
                                       `gtctATGAGCGTTTAAAGAACGACCGTGCTCCCTATGAGACACGCGCTCAGAATTGCGCTCAATATACCATCCCATCATTGTTCCCTAAGGACTCCGATAACGCCTCTACAGATTATCAAACTCCGTGGCAAGCCGTGGGCGCTCGTGGTCTGAACAATCTAGCCTCTAAGCTCATGCTGGCTCTATTCCCTATGCAGACTTGGATGCGACTTACTATATCTGAATATGAAGCAAAGCAGTTACTGAGCGACCCCGATGAACTCGCTAAGGTCGATGAGGGCCtctcctgtctcttataca                                                                                                                                                                                                                                                                >  1:376881/1‑287 (MQ=255)`  
                                         `ctATGAGCGTTTAAAGAACGACCGTGCTCCCTATGAGACACGCGCTCAGAATTGCGCTCAATATACCATCCCATCATTGTTCCCTAAGGACTCCGATAACGCCTCTACAGATTATCAAACTCCGTGGCAAGCCGTGGGCGCTCGTGGTCTGAACAATCTAGCCTCTAAGCTCATGCTGGCTCTATTCCCTATGCAGACTTGGATGCGACTTACTATATCTGAATATGAAGCAAAGCAGTTACTGAGCGACCCCGATGAACTCGCTAAGGTCGATGAGGGccctgtctcttatacacatctc                                                                                                                                                                                                                                                              >  1:156735/1‑281 (MQ=255)`  
                                           `aTGAGCGTTTAAAGAACGACCGTGCTCCCTATGAGACACGCGCTCAGAATTGCGCTCAATATACCATCCCATCATTGTTCCCTAAGGACTCCGATAACGCCTCTACAGATTATCAAACTCCGTGGCAAGCCGTGGGCGCTCGTGGTCTGAACAATCTAGCCTCTAAGCTCATGCTGGCTCTATTCCCTATGCAGACTTGGATGCGACTTACTATATCTGAATATGAAGCAAAGCAGTTACTGAGCGACCCAGATGAACTCGCTAAGGTCGATGAGGGCCTCTCGATGGTAGAGCGTatcat                                                                                                                                                                                                                                                            <  1:360379/301‑1 (MQ=255)`  
                                           `aTGAGCGTTTAAAGAACGACCGTGCTCCCTATGAGACACGCGCTCAGAATTGCGCTCAATATACCATCCCATCATTGTTCCCTAAGGACTCAGATAACGCCTCTACAGATTATCAAACTCCGTGGCAAGCCGTGGGCGCTCGTGGTCTGAACAATCTAGCCTCTAAGCTCATGCTGGCTCTATTCCCTATGCAGACTTGGATGCGACTTACTATATCTGAATATGAAGCAAAGCAGTTACTGAGCGACCCCGATGAACTCGCTAAGGTAGATGAGGGCCTCTCGATGGTAGAGCGTatcat                                                                                                                                                                                                                                                            <  1:683240/301‑1 (MQ=255)`  
                                                  `tttAAAGAACGACCGTGCTCCCTATGAGACACGCGCTCAGAATTGCGCTCAATATACCATCCCATCATTGTTCCCTAAGGACTCCGATAACGCCTCTACAGATTATCAAACTCCGTGGCAAGCCGTGGGCGCTCGTGGTCTGAACAATCTAGCCTCTAAGCTCATGCTGGCTCTATTCCCTATGCAGACTTGGATGCGACTTACTATATCTGAATATGAAGCAAAGCAGTTACTGAGCTACCCCGATGAACTCGCTAAGGTCGATGAGGGCCTCTCGATGGTAGAGCGTATCATCATGAAc                                                                                                                                                                                                                                                     <  1:660705/301‑1 (MQ=255)`  
                                                      `ggggctcggagatgtgtataagagaCA‑GCGCTTAAAATTGCGCTCAATATACCATCCCATCATTGTTCCCTAAGGACTCCGATAACGCCCCTACAGATTATCAAACTCCGTGGCAAGCCGTGGGCGCCCGTGGTCTGAACAATCTAGCCTCTAAGCTCATGCTGGCTCTATTCCCTATGCAGACTTGGATGCGACTTACTAAATCTGAATATGAAGCAAAGCAGTTACTGAGCGACCCCGATGAACTCGCTAAGGGCGATGAGGGCCTCTCGATGGTAGAGCGTATCATCATGAACTACAt                                                                                                                                                                                                                                                <  1:584863/280‑1 (MQ=255)`  
                                                      `aaGAACGACCGTGCTCCCTATGAGACACGCGCTCAGAATTGCGCTCAATATACCATCCCATCATTGTTCCCTAAGGACTCCGATAACGCCTCTACAGATTATCAAACTCCGTGGCAAGCCGTGTGCGCTCGTGGTCTGAACAATCTAGCCTCTAAGCTCATGCTGGCTCTATTCCCTATGCAGACTTAGATGCGACTTACTATATCTGAATATGAAGCAAAGCAGTTACTGAGCGACCCCGATGAACTCGCTAAGGTCGATGAGGTCCTCTCCATGGTAGAGCGTATCATCATGAACTACa                                                                                                                                                                                                                                                 >  1:163470/1‑301 (MQ=255)`  
                                                        `gAACGACCGTGCTCCCTATGAGACACGCGCTCAGAATTGCGCTCAATATACCATCCCATCATTGTTCCCTAAGGACTCCGATAACGCCTCTACAGATTATCAAACTCCGTGGCAAGCCGTTGGCGCTCGTTGTCTGAACAATCTAGCCTCTAAGCTCATGCTGGCTCTATTCCCTATGCAGACTTGTATGCGACTTACTATATCTGAATATGAAGCAAAGCAGTTACTGAGCGACCCCGATGAACTCGCTAAGGTCGATGAGGGCCTCTCGATGGTAGAGCGTATCATCATGAACTCCAtt                                                                                                                                                                                                                                               >  1:495955/1‑301 (MQ=255)`  
                                                             `aCCGTGCTACCTATGAGACACGCGAGCAGAATTGCGCTCAATATACCATCCCATCATTGTTCCCTAAGGACTACGATAACGCCTATACAGATTATCAAACTCCGTGGCAAGCCGTGGGCGCTCGTGGTCTGAACAATCTAGCCTCTAAGCTCATGCTGGCTCTATTCCCTATGCAGACTTGGATGCGACTTACTATATCTGAATATGAAGCAAAGCAGTGACTGAGCGACCCCGATGAACTCGCTAAGGTCGATGATGGCCTCTCGATGGTAGAGCGTATCATCATGAACTACATTGAGTc                                                                                                                                                                                                                                          <  1:259455/301‑1 (MQ=255)`  
                                                                 `tGCTCCCTATGAGACACGCGCTCAGAATTGCGCTCAATATACCATCCCATCATTGTTCCCTAAGGACTCCGATAACGCCTCTACAGATTATCAAACTCCGTGGCAAGCCGTGGGCGCTCGTGGTCTGAACAATCTAGCCTCTAAGCTCATGCTGGCTCTATTCCCTATGCAGACTTGGATGCGACTTACTATATCTGAATATGAAGCAAAGCAGTTACTGAGCGACCCCGATGAACTCGCTAAGGTCGATGAGGGCCTCTCGATGGTAGAGCGTATCATCATGAACTACATTGAGTCTAAc                                                                                                                                                                                                                                      <  1:750211/301‑1 (MQ=255)`  
                                                                   `cggagatgtgtataagagccaggAATTGCGCTCAATATACCCTCCCATCATTGTTCCCTAAGGACTCCGATAACGCCTCTACAGATTATCAAACTCCGTGGCAAGCCGTGGGCGCTCGTGGTCTGAACAATCTAGCCTCTAAGCTCATGCTGGCTCTATTCCCTATGCAGACTTGGATGCGACTTACTATATCTGAATATGAAGCAAAGCAGTTACTGAGCGACCCCGATGAACTCGCTAAGGTCGATGAGGGCCTCTCGATGGTAGAGCGTATCATCATGAACTACATTGAGTCTAACAg                                                                                                                                                                                                                                    <  1:534998/279‑1 (MQ=255)`  
                                                                    `ccccTATGAGACACGCGCTCAGAATTGCGCTCAATATACCATCCCATCATTGTTCCCTAAGGACTCCGATAACGCCTCTACAGATTATCAAACTCAGTGGCAAGCCGTGGGCGCTCGTGGTCTGAACAATCTAGCCTCTAAGCTCATGCTGGCTCTATTCCCTATGCAGACTTGGATGCGACTTACTATATCTGAATATGAAGCAAAGCAGTTACTGAGCGACCCCGATGAACTCGCTAAGGTCGATAAGGGCCTCTCGATGGTAGAGCGTATCATCATGAACTACATTGAGTCTAACAGt                                                                                                                                                                                                                                   <  1:521114/300‑1 (MQ=255)`  
                                                                     `cccTATGAGACACGCGCTCAGAATTGCGCTCAATATACCATCCCATCATTGTTCCCTAAGGACTCCGATAACGCCTCTACAGATTATCAAACTCCGTGGCAAGCCGTGGGCGCTCGTGGTCTGAACAATCTAGCCTCTAAGCTCATGCTGGCTCTATTCCCTATGCAGACTTGGATGCGACTTACTATATCTGAATATGAAGCAAAGCAGTTACTGAGCGACCCCGATGAACTCGCTAAGGTCGATGAGGGCCTCTCGATGGTAGAGCGTATCATCATGAACTACATTGAGTCTAACAGtt                                                                                                                                                                                                                                  >  1:543224/1‑301 (MQ=255)`  
                                                                     `cccTATGAGACACGCGCTCAGAATTGCGCTAAATATACCATCCCATCATTGTTCCCTAAGGACTCCGATAACGCCTCTACAGATTATCAAACTCCGTGGCAAGCCGTGGGAGCTCGTGGTCTGAACAATCTAGCCTCTAAGCTCATGCTGGCTCTATTCACTATGCAGACTTGGATGCGACTTACTATATCTGAATATGAAGCAAAGCAGTTACTGAGCGACCCCGATGAACTCGCTAAGGTCGATGAGGGCCTCTCGATGGTAGAGCGTATCATCATGAACTACATTGAGTCTAACAGtt                                                                                                                                                                                                                                  >  1:687242/1‑301 (MQ=255)`  
                                                                       `tgggctcggagatgtgtataagagacagcAATATACCATCCCATCATTGTTCCCTAAGGACTCCGATAACGCCTCTACAGATTATCAAACTCCGTGGCAAGCCGTGGGCTCTCGTGGTCTGAACAATCTAGCCTCTAAGCTCATGCTGGCTCTATTCCCTATGCAGACTTGGATGCGACTTACTATATCTGAATATGAAGCAAAGCAGTTACTGAGCGACCACGATGAACTCGCTAAGGTCGATGAGGGCCTCTCGATGGTAGAGCGTATCATCATGAACTACATTGAGTCTAACAGTTAc                                                                                                                                                                                                                                <  1:20082/273‑1 (MQ=255)`  
                                                                       `cTATGAGACACGCGCTCAGAATTGCGCTCAATATACCATCCCATCATTGTTCCCTAAGGACTCCGATAACGCCTCTACAGATTATCAAACTCCGTGGCAAGCCGTGGGCGCTCGTGGTCTGAACAATCTAGCCTCTAAGCTCATGCTGGCTCTATTCCCTATGCAGACTTGGATGCGACTTACTATATCTGAATATGAAGCAAAGCAGTTACTGAGCGACCCCGATGAACTCGCTAAGGTCGATGAGGGCCTCTCGATGGTAGAGCGTATCATCATGAACTACATTGAGTCTAACAGTTAc                                                                                                                                                                                                                                <  1:4521/301‑1 (MQ=255)`  
                                                                             `gaCACGCGCTCAGAATTGCGCTCAATATACCATCCCATCATTGTTCCCTAAGGACTCCGATAACGCCTCTACAGATTATCAAACTCCGTGGCAAGCCGTGGGCGCTCGTGGTCTGAACAATCTAGCCTCTAAGCTCATGCTGGCTCTATTCCCTATGCAGACTTGGATGCGACTTACTATATCTGAATATGAAGCAAAGCAGTTACTGAGCGACCCCGATGAACTCGCAAAGGTCGATGAGGGCCTCTCGATGGTAGAGCGTATCATCATGAACTACATTGAGTCTAACAGTTACCGAGTg                                                                                                                                                                                                                          >  1:447509/1‑301 (MQ=255)`  
                                                                               `cacGCGCTCAGAATTGCGCTAAATATACCATCCCATCATTGTTCCCTAAGGACTCCGATAAAGCCTCTACAGATTATCAAACTCCGTGGTAAGCCGTGGGCGCTCGTGGTCTGAACAATCTAGCCTCTAAGCTCATGCTGGCTCTATTCCCTATGCAGACTTGGAAGCGACTTACTATATCTGAATATGAAGCAAAGCAGTTACTGAGCGACCCCGATGAACTCGCTAAGGTCGATGAGGGCCTCTCGATGGTAGAGCGTATCATCATGAACTACATTGAGTCTAACAGTTACCGCGTgtc                                                                                                                                                                                                                        <  1:462306/301‑3 (MQ=255)`  
                                                                                         `gAATTGCGCTCAATATACCATCCCATCATTGTTCCCTAAGTACTCCGATAACGCCTCTACAGATTATCAAACTCCGTGGCAAGCCGTGGGCGCTCGTGGTCTGAACAATCTAGCCTCTAAGCTCATGCTGGCTCTATTCCCTATGCAGACTTGGATGCGACTTACTATATCTGAATATGAAGCAAAGCAGTTACTGAGCGACCCCGATGAACTCGCTAATGTCGATGAGGGCCTCTCGATGGTAGAGCGTATGATCATGAACTACATTGAGTCTAAAAGTTACCGCGTGActtgtctcttt                                                                                                                                                                                                              >  1:547303/1‑292 (MQ=255)`  
                                                                                         `gAATTGCGCTCAATATACCATCCCATCATTGTTCCCTAAGGACTCCGATAACGCCTCTACAGATTATCAAACTCCGTGGCAAGCCGTGGGCGCTCGTGGTCTGAACAATCTAGCCTCTAAGCTCATGCTGGCTCTATTCCCTATGCAGACTTGGATGCGACTTACTATATCTGAATATGCAGCAAAGCAGTTACTGAGCTACCCCGATGAACTCGCTAAGGTCGATGAGGGCCTCTAGATGGTAGAGCGTATCATCATGAACTACATTGAGTCTAACAGTTACCGCGTGACTCTCTTTGAg                                                                                                                                                                                                              >  1:231322/1‑301 (MQ=255)`  
                                                                                           `agagaaagaaaTATACCATCCCATCATTGTTCCCTAAGGACTCCGATAACGCCTCTACAGATTATCAAACTCCGTGGCAAGCCGTGGGCGCTCGTGGTCTGAACAATCTAGCCTCTAAGCTCATGCTGGCTCTATTCCCTATGCAGACTTGGATGCGACTTACTATATCTGAATATGAAGCAAAGCAGTTACTGAGCGACCCCGATGAACTCGCTAAGGTCGATGAGGGCCTCTCGATGGTAGAGCGTATCATCATGAACTACATTGAGTCTAACAGTTACCGCGTGACTCTCTTTGAGGc                                                                                                                                                                                                            <  1:303400/292‑1 (MQ=255)`  
                                                                                                   `cAATATACCATCCCATCATTGTTCCCTAATGACTCCGATAACGCCTCTACAGATTATCGAACTCCGTGGCAAGCCGTGGTCGCTCGTGGTCTGAACAATCTAGCCTCTAAGCTTATGCTGGCTCTATTCCCTATGCAGACTTGGATGCGACTTACTATATCTGTATATGAAGTAAAGCTGTTACTGAGCGACCCCTATGATCTCCCTAAGGTCGATGACGGCCTCTCGATAGTCGTGCGTATCATTATGAACTACACTGAGTCTAACAGTTACCGCCTGACTCTCTTTGTGGTTCTTAAAc                                                                                                                                                                                                    >  1:707190/1‑301 (MQ=255)`  
                                                                                                   `cAATATACCATCCCATCATTGTTCCCTAAGGACTCCTATAACGCCTCTACAGATTATCAAACTCCGTGGCAAGCCGTGGGCGCTCGTGGTCTGAACAATCTAGCCTCTAAGCTCATGCTGGCTCTATTCCCTATGCAGACTTGGATGCGACTAACTATATCTGAATATGAAGCAAAGCAGTTACTGAGCGACCCCGATGAACTCGCTAAGGTCGATGAGGGCCTCTCGATGGTAGAGCGTATCATCATGAACTACATTGAGTCTAACAGTTACCGCGTGACTCTCTTTGAGGCTCTCAAAc                                                                                                                                                                                                    >  1:285669/1‑301 (MQ=255)`  
                                                                                                   `cAATATACCATCCCATCATTGTTCCCTAAGGACTCCGATAACGCCTCTACAGATTATCAAACTCCGTGGCAAGCCGTGGGCGCTCGTGGTCTGAACAATCTAGCCTCTAAGCTCATTCTGGCTCTATTCCCTATGCAGACTTGGATGCGACTTACTATATCTGAATATGAAGCAAAGCAGTTACTGAGCGACCCCGATGAACTCGCTAAGGTCGATGAGGGCCTCTCGATGGTAGAGCGTATCATCATGAACTACATTGAGTCTAACAGTTACcgcgctgtctcttatacacatctccgag                                                                                                                                                                                                    >  1:771255/1‑277 (MQ=255)`  
                                                                                                   `cAATATACCATCCCATCATTGTTCCCTAAGGACTCCGATAACGCCTCTACAGATTATCAAACTCCGTGGCAAGCCGTGGGCGCTCGTGGTCTGAACAATCTAGCCTCTAAGCTCATGCTGGCTCTATTCCCTATGCAGACTTGGATGCGACTTACTATATCTGAATATGAAGCAAAGCAGTTACTGAGCGACCCCGATGAACTCGCTAAGGTCGATGAGGGCCTCTCGATTGTAGAGCATATCATCATGAACTACATTGAGTCTAACAGTTACCGCGTGACTCTCTTTGAGGCTCTCAAAc                                                                                                                                                                                                    <  1:144206/301‑1 (MQ=255)`  
                                                                                                   `cAATATACCATCCCATCATTGTTCCCTAAGGACTCCGATAACGCCTCTACAGATTATCAAACTCCGTGGCAAGCCGTGGGCGCTCGTGGTCTGAACAATCTAGCCTCTAAGCTCATGCTGGCTCTATTCCCTATGCAGACTTGGATGCGACTTACTATATCTGAATATGAAGCAAAGCAGTTACTGAGCGACCCCGATGAACTCGCTAAGGTCGATGAGGGCCTCTCGATGGTAGAGCGTATCATCATGAACTACATTGAGTCTAACAGTTACCGCGTGACTCTCTTTGAGGCTCTCAAAc                                                                                                                                                                                                    >  1:314118/1‑301 (MQ=255)`  
                                                                                                     `gatgtgtataagagacaggTTCCCTAAGGACTCCGATAACGCCTCTACAGATTATCAAACTCCGTGGCAAGCCGTGGGCGCTCGTGGTCTGAACAATCTAGCCTCTAAGCTCATGCTGGCTCTATTCCCTATGCAGACTTGGATGCGACTTACTATATCTGAATATGAAGCAAAGCAGTTACTGAGCGACCCCGATGAACTCGCTAAGGTCGATGAGGGCCTCTCGATGGTAGAGCGTATCATCATGAACTACATTGAGTCTAACAGTTACCGCGTGACTCTCTTTGAGGCTCTCAAACAg                                                                                                                                                                                                  <  1:246788/283‑1 (MQ=255)`  
                                                                                                     `cgtgtgctcggagatgtgtataagagacagcTCCGATAACGCCTCTACAGATTATCAAACTCCGTGGCAAGCCGTGGGCGCTCGTGGTCTGAACAATCTAGCCTCTAAGCTCATGCTGGCTCTATTCCCTATGCAGACTTGGATGCGACTTACTATATCTGAATATGAAGCAAAGCAGTTACTGAGCGACCCCGATGAACTAGCTAAGGTCGATGAGGGCCTCTCGATGGTAGAGCGTATCATCATGAACTACATTGAGTCTAACAGTTACCGCGTGACTCTCTTTGAGGCTCTAAAACAg                                                                                                                                                                                                  <  1:168435/271‑1 (MQ=255)`  
                                                                                                     `atatACCATCCCATCATTGTTCCCTAAGGACTCCGATAACGCCTCTACAGATTATCAAACTCCGTGGCAAGCCGTGGGCGCTCGTGGTCTGAACAATCTAGCCTCTAAGCTCATGCTGGCTCTATTCCCTATGCAGACTTGGATGCGACTTACTATATCTGAATATGAAGCAAAGCAGTTACTGAGCGACCCCGATGAACTCGCTAAGGTCGATGAGGGCCTCTCGATGGTAGAGCGTATCATCATGAACTACATTGAGTCTAACAGTTACCGCGTGACTCTCTTTGAGGCTCTCAAACAg                                                                                                                                                                                                  >  1:185691/1‑301 (MQ=255)`  
                                                                                                            `atcccatcATTGTTCCCTAAGGACTCCGATAACGCCTCTACAGATTATCAAACTCCGTGGCAAGCCGTGGGCGCTCGTGGTCTGAACAATCTAGCCTCTAAGCTCATGCTGGCTTTATTCCCTATGCAGACTTGGATGCGACTTACTATATCTGAATATGAAGCAAAGCAGTTACTGAGCGACCCCGATGAACTCGCTAAGGTCGAAGAGGGCCTCTCGATGGTAGAGCGTATCATCATTAACTACATTGAGTCTAACAGTTACCGCGTGACTCTCTTTGAGGCTCTCAAACAGTTAGTCg                                                                                                                                                                                           <  1:92238/301‑1 (MQ=255)`  
                                                                                                             `tcccatcATTGTTCCCTAAGGACTCCGATAACGCCTCTACAGATTATCAAACTCCGTGGCAAGCCGTGGGCGCTCGTGGTCTGAACAATCTAGCCTCTAAGCTCATGCTGGCTCTATTCCCTATGCAGACTTGGATGCGACTTACTATATCTGAATATGAAGCAAAGCAGTTACTGAGCGACCCCGATGAACTCGCTAAGGTCGATGAGGGCCTCTCGATGGTAGAGCGTATCATCATGAACTACATTGAGTCTAACAGTTACCGCGTGACCCTCTTTGAAGCTCTCAAACAGTTAGTCGt                                                                                                                                                                                          >  1:406518/1‑301 (MQ=255)`  
                                                                                                              `cccatcATTGTTCCCTAAGGACTCCGATAACGCCTCTACAGATTATCAAACTCCGTGGCAAGCCGTGGGCGCTCGTGGTCTGAACAATCTAGCCTCTAAGCTCATGCTGGCTCTATTCCATATGCAGACTTGGATGCGACTTACTATATCTGAATATGAAGCAAAGCAGTTACTGAGCGACCCCGATGAACTCGCTAAGGTCGATGAGGGCCTCTCGATGGTAGAGCGTATCATCATGAACTACATTGAGTCTAACAGTTACCGCGTGACTCTCTTTGAGGCTCTCAAAAAGTTAGTCGTa                                                                                                                                                                                         >  1:387559/1‑301 (MQ=255)`  
                                                                                                                       `ggctcggagatgtgtataagagacagctACAGATTATCAAACTCCGTGGCAAGCCGTGGGCGCTCGTGGTCTGAACAATCTAGCCTCTAAGCTCATGCTGGCTCTATTCCCTATGCAGACTTGGATGCGACTTACTATATCTGAATATGAAGCAAAGCAGTTATTGAGCGACCCCGATGAACTCGCTAAGGTCGATGAGGGCCTCTCGATGGTAGAGCGTATCATCATGAACTACATTGAGTCTAACAGTTACCGCGTGACTCTCTTTGAGGCTCTCAAACAGTTAGTCGTAGCTGGTAAc                                                                                                                                                                                <  1:336989/275‑1 (MQ=255)`  
                                                                                                                       `gTTCCCTAAGGACTCCGATAACGCCTCTACAGATTATCAAACTCCGTTGCAAGCCGTGGGCGCTCGTGGTCTGAACAATCTAGCCTCTAAGCTCATGCTGGCTCTATTCCCTATGCAGACTTGGATGCGACTTACTATATCTGAATATGAAGCAAAGCAGTTACTGAGCGACCCCGATGAACTCGCTAAGGTCGATGAGTGCCTCTCGATGGTAGAGCGTATCATCATGAACTACATTGAGTCTAACAGTTACCGCGTGACTCTCTTTGAGGCTCTCAAACAGTTAGTCGTAGCTGGTAAc                                                                                                                                                                                <  1:639763/301‑1 (MQ=255)`  
                                                                                                                       `gTTCCCTAAGGACTCCAATAACGCCTCTACAGATTATCAAACTCCGTGGCAAGCCGTGGGCGCTCGTGGTCTGAACAATCTAGCCTCTAAGCTCATGCTGGCTCTATTCCCTATGCAGACTTGGATGCGACTTACTATATCTGAATATGAAGCAAAGCAGTTACTGAGCGACCCCGATGAACTCGCTAAGGTCGATGAGGGCCTCTCGATGGTAGAGCGTATCATCATGAACTACATTGAGTCTAACAGTTACCGCGTGACTCTCTTTGAGGCTCTCAAACAGTTAGTCGTAGCTGGTAAc                                                                                                                                                                                >  1:101550/1‑301 (MQ=255)`  
                                                                                                                           `cggagatgtgtataagagacagctACAGATTATCAAACTCCGTGGCAAGCCGTGGGCGCTCGTGGTCTGAACAATCTAGCCTCTAAGCTCATGCTGGCTCTATTCCCTATGCAGACTTGGATGCGACTTACTATATCTGAATATGAAGCAAAGCAGTTACTGAGCGACCCCGATGAACTCGCTAAGGTCGATGAGGGCCTCTCGATGGTAGAGCGTATCATCATGAACTACATTGAGTCTAACAGTTACCGCGTGACTCTCTTTGAGGCTCTCAAACAGTTAGTCGTAGCTGGTAACGTcc                                                                                                                                                                            <  1:731020/279‑1 (MQ=255)`  
                                                                                                                                        `aTAACGCCTCTACAGATTATCAAACTCCGTGGCAAGCCGTGGGCGCTCGTGGTCTGAACAATCTAGCCTCTAAGCTCATGCTGGCTCTATTCCCTATGCAGACTTGGATGCGACTTACTATATCTGAATATGAAGCAAAGCAGTTACTGAGCGACCCCGATGAACTCGCTAAGGTCGATGAGGGCTTCTCGATGGTAGAGCGTATCATCATGAACTACATTGAGTCTAACAGTTACCGCGTGACTCTCTTTGAGGCTCTCAAACAGTTAGTCGTAGCTGGTAACGTCCTGCTGtacctacc                                                                                                                                                               <  1:593092/301‑1 (MQ=255)`  
                                                                                                                                            `cGCCTCTACAGATTATCAAACTCCGTGGCAAGCCGTGGGCGCTCGTGGTCTGAACAATCTAGCCTCTAAGCTCATGCTGGCTCTATTCCCTATGCAGACTTGGATGCGACTTACTATATCTGAATATGAAGCAAAGCAGTTACTGAGCGACCCCGATGAACTCGCTAATGTCGATGAGGGCCTCTCGATGGTAGAGCGTATCATCATGAACTACATTGAGTCTAACAGTTACCGCGTGACTCTCTTTGAGGCTCTCAAACAGTTAGTCGTAGCTGGTAACGTCCTGCTGtacctaccctgt                                                                                                                                                           >  1:422347/1‑297 (MQ=255)`  
                                                                                                                                                      `gATTATCAAACTCCGTGGCAAGCCGTGGGCGCTCGTGGTCTGAACAATCTAGCCTCTAAGCTCATGCTGGCTCTATTCCCTATGCAGACTTGGATGCGACTTACTATATCTGAATATGAAGCAAAGCAGTTACTGAGCGACCCCGATGAACTCGCTAATGTCGATGAGGGCCTCTCGATGGTATAGCGTATCATCATGAACTACATTGAGTCTAACAGTCACCGCGTGACTCTCTTTGAGGCTCTCAAACAGTTAGTCGTAGCTGGTAACGTCCTGCTGTACCTACCGGAACCGGAAGGGt                                                                                                                                                 >  1:716438/1‑301 (MQ=255)`  
                                                                                                                                                               `aCTCCGTGGCAAGCCGTGGGCGCTCGTGGTCTGTACAATCTAGCCTCTAAGCTCATGCTGGCTCTATTCCATATGCAGACTTGAATGCGACTTACTATATCTGAATATGAAGCAAAGCAGTTACTGAGCGACCCCGATGAACTCGCTAAGGTCGATGAGGGCCTCTCGATGGTAGAGCGTATCATCATGAACTACATTGAGTCTAACAGTTACCGCGTGACTCTCTTTGAGGCTCTCAAACAGTTAGTCGTAGATGGTAACGTCCTGCTGTACCTACCGGAACCGGAAGGGTCAAACtata                                                                                                                                        >  1:223326/1‑301 (MQ=255)`  
                                                                                                                                                                `cTCCGTGGCAAGCCGTGGGCGCTCGTGGTCTGAACAATCTAGCCTCTAAGCTCATGCTGGCTCTATTCCCTATGCAGACTTGGATGCGACTTACTATATCTGAATATGAAGCAAAGCAGTTACTGAGCGACCCCGATGAACTCGCTAAGGTCGATGAGGGCCTCTCGTTGGTAGAGCGTATCATCATGAACTACATTGAGTCTAACAGTTACCGCGTGACTCTCTTTGAGGCTCTCAGACAGTTAGTCGTAGCTGGTAACGTCCTGCTGtacctctgtctcttatacccatctccgacccc                                                                                                                                       >  1:790579/1‑274 (MQ=255)`  
                                                                                                                                                                   `cGTGGCAAGCCGTGGGCGCTCGTGGTCTGAACAATCTAGCCTCTAAGCTCATGCTGGCTCTATTCCCTATGCAGACTTGGATGCGACTTACTATATCTGAATATGAAGCAAAGCAGTTACTGAGCGACCCCGATGAACTCGCTAAGGTCGATGAGGGCCTCTCGATGGTAGAGCGTATCATCATGAACTACATTGAGTCTAACAGTTACCGCGTGACTCTCTTTGAGGCTCTCAAACAGTTAGTCGTAGCTGGTAACGTCCTGCTGTACCTACCGGAACCGGAAGGGTCAAACTATAATcc                                                                                                                                    <  1:241410/301‑1 (MQ=255)`  
                                                                                                                                                                   `cGTGGCAAGCCGTGGGCGCTCGTGGTCTGAACAATCTAGCCTCTAAGCTCATGCTGGCTCTATTCCCTATGCAGACTTGGATGCGACTTACTATATCTGAATATGAAGCAAAGCAGTTACTGAGCGACACCGATGAACTCGCTAAGGTCGATGAGGGCCTCTCGATGGTAGAGCGTATCATCATGAACTACATTGAGTCTAACAGTTACCGCGTGACTCTCTTTGAGGCTCTCAAACAGTTAGTCGTAGCTGGTAACGTCCTGCTGTACCTACCGGAACCGGAAGGGTCAAACTATAATcc                                                                                                                                    <  1:530013/301‑1 (MQ=255)`  
                                                                                                                                                                           `gCCGTGGGCGCTCGTGGTCTGAACAATCTAGCCTCTAAGCTCATGCTGGCTATATTCACTATGCAGACTTGGATGCGACTTACTATATCTGAATATGAAGCAAAGCAGTTACTGAGCGACACCGATGAACTAGCTAAGGTCGATGAGGGCCTCTCGATGGTAGAGCGTATCATCATGAACTAAATAGAGTCTAACAGTTACCGCGTGACTCTCTTTGAGGCTCTCAAACAGTTAGTCGTAGCAGGTAACGTCCTGCTGTACCTACCGGAACCGGAAGGGTCAAACTATAATCCCATGAAGc                                                                                                                            <  1:131805/301‑1 (MQ=255)`  
                                                                                                                                                                                `gggCGCTCGTGTTCTGAACAATCTAGCCTCTAAGCTCATGCTGGCTCTATTCCCTATGCAGACTTGGATGCGACTTACTATATCTGAATATGAAGCAAAGCAGTTACTGAGCGACCCCGATGAACTCGCTAAGGTCGATGAGGGCCTCTCGATGGTAGAGCGTATCATCATGAACTACATTGAGTCTAACAGTTACCGCGTGACTCTCTTTGAGGCTCTCAAACAGTTAGTCGTAGCTGGTAACGTCCTGCTGTACCTACCGGAACCGGAAGGGTCAAACTATAATCCCATGAAGCTGTAc                                                                                                                       <  1:738691/301‑1 (MQ=255)`  
                                                                                                                                                                                     `tcgtagatgtgtataagagacagtctAAGCTCCAGCTGGCTCTATTCCCTATGCAGACTTGGATTCGACTTACTATATCTGAATATGAAGCAAAGCAGTTACTGAGCGACCCCGATGAACTCGCTAAGGTCGATGAGGGCCTCTCGATGGTAGAGCGTATCATCATGAACTACATTGAGTCTAACAGTTACCGCGTGACTCTCTTTGAGGCTCTCAAACAGTTAGTCGTAGCTGGTAACGTCCTGCTGTACCTACCGGAACCGGAAGGGTCAAACTATAATCCCATGAAGCTGTACCGAtt                                                                                                                  <  1:116720/278‑1 (MQ=255)`  
                                                                                                                                                                                     `ctagTGGTCTGAACAATCTAGCCTCTAAGCTCATGCTGGCTCTATTCCCTATGCAGACTTGGATGCGACTTACTATATCTGAATATGAAGCAAAGCAGTTACTGAGCGACCCCGATGAACTCGCTAAGGTCGATGAGGGCCTCTCGATGGTAGAGCGTATCATCATGAACTACATTGAGTCTAACAGTTACCGCGTGACTCTCTTTGAGGCTCTCAAACAGTTAGTCGTAGCTGGTAACGTCATGCTGTACCTACCGGAACCGGAAGGGTCAAACTATAATCCCATGAAGCTGTACCGAtt                                                                                                                  <  1:355200/298‑1 (MQ=255)`  
                                                                                                                                                                                     `cTCGTCGTCTGAACAATCTAGCCTCTAAGCTCATGCTGGCTCTATTCCCTATGCAGACTTGGATGCGACTTACTATATCTGAATATGAAGCAAAGCAGTTACTGAGCGACCCCGATGAACTAGCTAAGGTCGATGAGGGCCTCTCGATGGTAGAGCGTATCATCATGAACTACATTGAGTCTAACAGTTACCGCGTGACTCTCTTTGAGGCTCTCAAACAGTTAGTCGTAGCTGGTAACGTCCTGCTGTACCTACCGGAACCGGAAGGGTCAAACTATAATCCCATGAAGCTGTAcctgtc                                                                                                                  >  1:655455/1‑297 (MQ=255)`  
                                                                                                                                                                                              `tGAACAATCTAGCCTCTAAGCTCATGCTGGCTCTATTCCCTATGCAGACTTGGATGCGACTTACTATATCTGAATATGAAGCAAAGCAGTTACTGAGCGACCCCGATGAACTCGCTAAGGTCGATGAGGGCCTCTCGATGGTAGAGCGTATCATCATGAACTACATTGAGTCTAACAGTTACCGCGTGACTCTCTTTGAGGCTCTCAAACAGTTAGTCGTAGCTGGTAACGTCCTGCTGTACCTACCGGAACCGTAAGGGTCAAACTATAATCCCATGAAGCTGTACCGATTGTCTTCTTa                                                                                                         <  1:610048/301‑1 (MQ=255)`  
                                                                                                                                                                                                            `tctAAGCTCATGCTGGCTCTATTCCCTATGCAGACTTGGATGCGACTTACTATATCTGAATATGAAGCAAAGCAGTTACTGAGCGACCCCGATGAACTCGCTAAGGTCGATGAGGGCCTCTCGATGGTAGAGCGTATCATCATGAACTACATTGAGTCTAACAGTTACCGCGTGACTCTCTTTGAGGCTCTCAAACAGTTAGTCGTAGCTGGTAACGTCCTGCTGTACCTACCGGAACCGGAAGGGTCAAACTATAATCCCATGAAGCTGTACCGATTGTCTTCTTATGTGGTCCAAagag                                                                                           <  1:668307/301‑1 (MQ=255)`  
                                                                                                                                                                                                            `tctAAGCTCATGCTGGCTCTATTCCCTATGCAGACTTGGATGCGACTTACTATATCTGAATATGAAGCAAAGCAGTTACTGAGCGACCCCGATGAACTCGCTAAGGTCGATGAGGGCCTCTCGATGGTAGAGCGTATCATCATGAACTACATTGAGTCTAACAGTTACCGCGTGACTCTCTTTGAGGCTCTCAAACAGTTAGTCGTAGCTGGTAACGTCCTGCTGTACCTACCGGAACCGGAAGGGTCAAACTATAATCCAATGAAGCTGTACCGATTGTCTTCTTATGTGGTCCAACgag                                                                                           >  1:367097/1‑301 (MQ=255)`  
                                                                                                                                                                                                                `aGCTCATGCTGGCTCTATTCCCTATGCAGACTTGGATGCGACTTACTATATCTGAATATGAAGCAAAGCAGTTACTGAGCGACCCCGATGAACTCGCTAAGGTCGATGAGGGCCTCTCGATGGTAGAGCGTATCATCATGAACTACATTGAGTCTAACAGTTACCGCGTGACTCTCTTTGAGGCTCTCAAACAGTTAGTCGTAGCTGGTAACGTCCTGCTGTACCTACCGGAACCGGAAGGGTCAAACTATAATCCCATGAAGCTGTACCGATTGTATTCTTactgtctcttatacacatc                                                                                       >  1:412092/1‑283 (MQ=255)`  
                                                                                                                                                                                                                    `cATGCTGGCTCTATTCCCTATGCAGACTTGGATGCGACTTACTATATCTGAATATGAAGCAAAGCAGTTACTGAGCGACCCCGATGAACTCGCTAAGGTCGATGAGGGCCTCTCGATGGTAGAGCGTATCATCATGAACTACATTGAGTCTAACAGTTACCGCGTGACTCTCTTTTAGGCTCTCAAACAGTTAGTCGTAGCTGGTAACGTCCTGCTGTACCTACCGGAACCGGAAGGGTCAAACTATAATCCCATGAAGCTGTACCGATTGTCTTCTTATGTGGTCCACCGAGACGCATTc                                                                                   >  1:797346/1‑301 (MQ=255)`  
                                                                                                                                                                                                                                   `cccTATGCAGACTTGGATGCGACTTACTATATCTGAATATGAAGCAAAGCAGTTACTTAGCGACCCCGATGAACTCGCTAAGGTCGATGAGGGCCTCTCGATGGTAGAGCGTATCATCATGAACTACATTGAGTCTACCAGTTACCGCGTGACTCTCTTTGAGGCTCTCAAACAGTTAGTCGTAGCTGGTAACGTCCTGCTGTACCTACCGGAACCGGAAGGGTCAAACTATAATCCCATGAAGCTGTACCGCTTGTCTTCTTATGTGGTCCAACGAGACGCATTCGGCAACGTTCTGCaa                                                                    >  1:318960/1‑301 (MQ=255)`  
                                                                                                                                                                                                                                   `cccTATGCAGACTTGGATGCGACTTACTATATCTGAATATGAAGCAAAGCAGTTACTGAGCGACCCCGATGAACTCGCTAAGGTCGATGAGGGCCTCTCGATGGTAGAGCGTATCATCATGAACTACATTGAGTCTAACAGTTACCGCGTGACTCTCTTTGAGGCTCTCAAACAGTTAGTCGTAGCTGGTAACGTCCTGCTGTACCTACCGGAACCGGAAGGGTCAAACTATAATCCCATGAAGCTGTACCGATTGTCTTCTTATGTGGTCCAACGAGACGCATTCGGCAACGTTCTGCaa                                                                    >  1:573314/1‑301 (MQ=255)`  
                                                                                                                                                                                                                                   `cccTATGCAGACTTGGATGCGACTTACTATATCTGAATATGAAGCAAAGCAGTTACTGAGCGACCCCGATGAACTCGCTAAGGTCGATGAGGGCCTCTCGATGGTAGAGCGTATCATCATGAACTACATTGAGTCTAACAGTTACCGCGTGACTCTCTTTGAGGCTCTCAAACAGTTAGTCGTAGCTGGTAACGTCCTGCTGTACCTACCGGAACCGGAAGGGTCAAACTATAATCCAATGAAGCTGTACCGATTGTCTTCTTATGTGGTCCAACGAGACGCATTCGGCAACGTTCTGCaa                                                                    <  1:381093/301‑1 (MQ=255)`  
                                                                                                                                                                                                                                    `ccTATGCAGACTTGGATGCGACTTACTATATCTGAATATGAAGCAAAGCAGTTACTGAGCGACCCCGATGAACTCGCTAAGGTAGATGAGGGCCTCTCGATGGTAGAGCGTATCATCATGAACTACATTGAGTCTAACAGTTACCGCGTGACTCTATTTGAGGCTCTCAAACCGTTAGTCGTAGCTGGTAACGTCCTGCTGTACCTACCGGAACCGGAAGGGTCAAACTATAATCCCATGAAGCTGTACCGATTGTCTTCTTATGTGGTCCAACGAGACGCATTCGTCAACGTTCTGCaaa                                                                   >  1:251080/1‑301 (MQ=255)`  
                                                                                                                                                                                                                                            `gACTTGGATGCGACTTACTATATCTGAATATGAAGCAAAGCAGTTACTGAGCGACCCCGATGAACTCGCTAAGGTCGATGGGGGCCTCTCGATGGTAGAGCGTATCATCATGAACTACATTGAGTCTAACAGTTACCACGTGACTCTCTTTGAGGCTCTCAAACAGTTAGTCGTAGCTGGTAACGTCCTGCTGTACCTACCTGAACCGGACTGGTCAAACTATAATCCCCTGAAGCTGTACCGATTGTCTTCTTATGTGGTCCAACGAGACGCATTCGGCAACGTTCTGCAAATGGTGACt                                                           >  1:326074/1‑301 (MQ=255)`  
                                                                                                                                                                                                                                            `gACTTGGATGCGACTTACTATATCTGAATATGAAGCAAAGCAGTTACTGAGCGACCCCGATGAACTCGCTAAGGTCGATGAGGGCCTCTCGATGGTAGAGCGTATCATCATGAACTACATTGAGTCTAACAGTTACCGCGTGACTATCTTTGAGGCTCTCAAACAGTTAGTCGTAGCTGGTAACGTCCTGCTGTACCTACCGGAACCGGAAGGGTCAAACTATAATCCCATGAAGCTGTACCGATTGTCTTCTTATGTGGTCCAACGAGACGCATTCGGCAACGTTCTGCAAATGGTGACt                                                           >  1:621637/1‑301 (MQ=255)`  
                                                                                                                                                                                                                                                 `ggATGCGACTTACTATATCTGAATATGAAGCAAAGCAGTTACTGAGCGACCCCGATGAACTCGCTAAGGTCGATGAGGGCCTCTCGATGGTAGAGCGTATCATCATGAACTACATTGAGTCTAACAGTTACCTCGTGACTCTCTTTGAGGCTCTCAAACAGTTAGTCGTAGCTGGTAACGTCCTGCTGTACCTACCGGAACCGGAAGGGTCAAACTATAATCCCATGAAGCTGTACCGATTGTCTTCTTATGTGGTCCAACGAGACGCATTCGGCAACGTTCTGCAAATggctgtctctta                                                      >  1:179588/1‑291 (MQ=255)`  
                                                                                                                                                                                                                                                   `ggtataagagacagatCTGAATATGAAGCAAAGCAGTTACTGAGCGACCCCGATGAACTCGCTAAGGTCGATGAGGGCCTCTCGATGGTAGAGCGTATCATCATGAACTACATTGAGTCTAACAGTTACCGCGTGACTCTCTTTGAGGCTCTCAAACAGTTAGTCGTAGCTGGTAACGTCCTGCTGTACCTACCGGAACCGGAAGGGTCAAACTATAATCACATGAAGCTGTACCGATTGTCTTCTTATGTGGTCCAACGAGACGCATTCGGCAACGTTCTGCAAATGGTAACTCGTGAcc                                                    <  1:279515/287‑1 (MQ=255)`  
                                                                                                                                                                                                                                                     `gCGACTTACTATATCTGAATATGAAGCAAAGCAGTTACTGAGCGACCCCGATGAACTCGCTAAGGTCGATGAGGGCCTCTCGATGGTAGAGCGTATCATCATGAACTACATTGAGTCTAACAGTTACCGCGTGACTCTCTTTGAGGCTCTCAAACAGTTAGTCGTAGCTGGTAACGTCCTGCTGTACCTACCGGAACCGGAAGGGTCAAACTATAATCCCATGAAGCTGTACCGATTGTCTTCTTATGTGGTCCAACGAGACGCATTCGGCAACGTTCTGCAAATGGTGACTCGTGACCAg                                                  <  1:507939/301‑1 (MQ=255)`  
                                                                                                                                                                                                                                                       `gACTTACTATATCTGAATATGAAGCAAAGCAGTTACTGAGCGACCCCGATGAACTCGCTAAGGTCGATGAGGGCCTCTCGATGGTAGAGCGTATCATCATGAACTACATTGAGTCTAACAGTTACCGCGTGACTCTCTTTGAGGCTCTCAAACAGTTAGTCGTAGCTGGTAACGTCCTGCTGTACCTACCGGAACCGGAAGGGTCAAACTATAATCCCATGAAGCTGTACCGATTGTCTTCTTATGTGGTCCAACGAGACGCCTTCGGCAACGTTCTGCCAATGGTTACTCGTGACCAGAt                                                >  1:512160/1‑301 (MQ=255)`  
                                                                                                                                                                                                                                                       `aaagaggcagaacTGAATATGAAGCAAAGCAGTTACTGAGCGACCCCGATGAACTCGCTAAGGTCGATGAGGGCCTCTCGATGGTAGAGCGTATCATCATGAACTACATTGAGTCTAACAGTTACCGCGTGACTCTCTTTGAGGCTCTCAAACAGTTAGTCGTAGCTGGTAACGTCCTGCTGTACCTACCGGAACCGGAAGGGTCAAACTATAATCCCATGAAGCTGTACCGATTGTCTTCTTATGTGGTCCAACGAGACGCATTCGGCAACGTTCTGCAAATGGTGACTAGTGACCAGAt                                                <  1:33349/289‑1 (MQ=255)`  
                                                                                                                                                                                                                                                         `gtgggctcggagatgtgtataagagacaggTTACTGAGCGACCCCGATGAACTCGCTAAGGTCGATGAGTGCCTCTCGATGGTAGAGCGTATCATCATGAACTACATTGAGTCTAACTGTTACCGCGTGACTCTCTTTGAGGCTCTCAAACAGTTAGTCGTAGCTGGTAACGTCCTGCTGTACCTACCGGAACCGGAAGGGTCAAACTATAATCCCATGAAGCTGTACCGATTGTCTTCTTATGTGGTCCAACGAGACGCATTCGGCAACGTTCTGCAAATGGTGACTCGTGACCAGATAg                                              <  1:606406/272‑1 (MQ=255)`  
                                                                                                                                                                                                                                                             `cgtagatgtgtataaggggcaggcaGTGACGGAGCGACCCCGATGAACTCGCTAAGGTCGATGAGGGCCTCTCGATGGTAGAGCGTATCATCATGAACTACATTGAGTCTAACAGTTACCGCGTGACTCTCTTTGAGGCTCTCAAACAGTTAGTCGTAGCTGGTAACGTCCTGCTGTACCTACCGGAACCGGAAGGGTCAAACTATAATCCCATGAAGCTGTACCGATTGTCTTCTTATGTGGTCCAACGAGACGCATTCGGCAACGTTCTGCAAATGGTGACTCGTGACCAGATAGCttt                                          <  1:85850/279‑1 (MQ=255)`  
                                                                                                                                                                                                                                                                 `atCTGAATATGAAGCAAAGCAGTTACTGAGCGACCCCGATGAACTCGCTAAGGTCGATTAGGGCCTCTCGATGGTAGAGCGTATCATCATGAACTACATTGAGTCTAACAGTTACCGCGTGACTTTCTTTGAGGCTCTCAAACAGTTAGTCGTAGCTGGTAACGTCCTGCTGTACCTACCGGAACCGGAAGGGTCAAACTATAATCCCATGAAGCTGTACCGATTGTCTTCTTATGTGGTCCAACGAGACGCATTCGGCAACGTTCTGCAAATGGTGACTCGTGACCAGATAGCTTTTTGt                                      >  1:577807/1‑301 (MQ=255)`  
                                                                                                                                                                                                                                                                     `gAATATGAAGCAAAGCAGTTACTGAGCGACCCCGATGAACTCGCTAAGGTCTATGAGGGCCTCTCGATGGTAGAGCGTATCATCATGAACTACATTGAGTCTAACAGTTACCGCGTGACTCTCTTTGAGGCTCTCAAACAGTTAGTCGTAGCTGGTAACGTCCTGCTGTACCTACCGGAACCGGAAGGGTCAAACTATAATCCCATGAAGCTGTACCGATTGTCTTCTTATGTGGTCCAACGAGACGCATTCGGCAACGTTCTGCAAATGGTGACTCGTGACCAGATAGCTTTTGGTGctc                                  <  1:777915/301‑1 (MQ=255)`  
                                                                                                                                                                                                                                                                       `atatGAAGCAAAGAAGTTACTGAGCGACCCCGATGAACTCGCTAAGGTCGATGAGGGCCTCTAGATGGTAGAGCGTATCATCATGAACTACATTGAGTCTAACAGTTACCGCGTGACTCTCTTTGAGGCTCTCAAACAGTTAGTCGTAGCTGGTAACGTCCTGCTGTACCTACCAGAACCGGAAGGGTCAAACTATAATCCCATGAAGCTGTACCGATTGTCTTCTTATGTGGTCCAACGAGACGCATTCGGCAACGTTCTGCAAATGGTGACTCGTGACCAGATAGCTTTTGGTGCtgtc                                >  1:309693/1‑301 (MQ=255)`  
                                                                                                                                                                                                                                                                        `aatGAAGCAAAGCAGTTACTGAGCGACCCCGATGAACTCGCTAAGGTCGATGAGGGCCTCTCGATGGTAGAGCGTATCATCATGAACTACATTGAGTCTAACAGTTACCGCGTGACTCTCTTTGAGGCTCTCAAACAGTTAGTCGTAGCTGGTAACGTCCTGCTGTACCTACCGGAACCGGAAGGGTCAAACTATAATCCCATGAAGCTGTACCGATTGTCTTCTTATGTGGTCCAACTAGACGCATTCGGCAACGTTCTGCAAATGGTGACTCGTGACCAGATAGCTTTTGGTGCTCTcc                               <  1:601976/300‑1 (MQ=255)`  
                                                                                                                                                                                                                                                                         `gtgtataagagacagtACTGAGCGACCCCGATGAACTCGCTAAGGTCGATGAGGGCCTCTCGATGGTAGAGCGTATCATCATGAACTACATTGAGTCTAACAGTTACCGCGTGACTCTCTTTGAGGCTCTCAAACAGTTAGTCGTAGCTGGTAACGTCCTGCTGTACCTACCGGAACCGGAAGGGTCAAACTATAATCCCATGAAGCTGTACCGATTGTCTTCTTATGTGGTCCAACGAGACGCATTCGGCAACGTTCTGCAAATGGTGACTCGTGACCAGATAGCTTTTGGTGCTCTccc                              <  1:298412/286‑1 (MQ=255)`  
                                                                                                                                                                                                                                                                            `aagcaaagcaGTTACTGAGCGACCCCGATGAACTCGCTAAGGTCGATGAGGGCCTCTCGATGGTAGATCGTATCATCATGAACTACATTGAGTCTAACAGTTACCGCGTGACTCTCTTTGAGGCTCTCAAACAGTTAGTCGTAGCTGGTAACGTCCTGCTGTACCTACCGGAACCGGAAGGGTCAAACTATAATCCCATGAAGCTGTACCGATTGTCTTCTTATGTGGTCCAACGAGACGCATTCGGCAACGTTCTGCAAATGGTGACTCGTGACCAGATAGCTTTTGGtctgtctcttat                           >  1:628713/1‑290 (MQ=255)`  
                                                                                                                                                                                                                                                                              `gcaaagcaGTTACTGAGCGACCCCGATGAACTCGCTAAGGTCGATGAGGGCCTCTCGATGGTAGAGCGTATCATCATGAACTACATTGAGTCTAACAGTTACCGCGTGACTCTCTTTGAGGCTCTCAAACAGTTAGTCGTAGCTGGTAACGTCCTGCTGTACCTACCGGAACCGGAATGGTCAAACTATAATCCCATGAAGCTGTACCGATTGTCTTCTTATGTGGTCCAACGAGACGCATTCGGCAACGTTCTGCAAATGGTGACTCGTGACCAGATAGCTTTTGGTGCTCTCCCTGAgg                         <  1:194082/301‑1 (MQ=255)`  
                                                                                                                                                                                                                                                                               `aatgtgtataagggacagACCCCGATGAACTCGCTAAGGTCGATGAGGGCCTCTCGATGGTAGAGCGTATCATCATGAACTACATTGAGTCTAACAGTTACCGCGTGACTCTCTTTGAGGCTCTCAAACAGTTAGTCGTAGCTGGTAACGTCCTGCTGTACCTACCGGAACCGGAAGGGTCAAACTATAATCCCATGAAGCTGTACCGATTGTCTTCTTATGTGGTCCAACGAGACGCATTCGGCAACGTTCTGCAAATGGTGACTCGTGACCAGATAGCTTTTGGTGCTCTCCCTGAGGa                        <  1:672349/284‑1 (MQ=255)`  
                                                                                                                                                                                                                                                                               `aaaagcaGTTACTGAGCGACCCCGATGAACTCGCTAAGGTCGATGAGGGCCTCTCGATGGTAGAGCGTATCATCATGAACTACATTGAGTCTAACAGTTACCGCGTGACTCTCTTTGAGGCTCTCAAACAGTTAGTCGTAGCTGGTAACGTCCTGCTGTACCTACTGGAACCGGAAGGGTCAAACTATAATCCCATGAAGCTGTACCGATTGTCTTCTTATGTGGTCCAACGAGACGCATTCGGCAACGTTCTGCAAATGGTGACTCGTGACCAGATAGCTTTTTGTGCTCTCCCTGAGGa                        <  1:457230/300‑1 (MQ=255)`  
                                                                                                                                                                                                                                                                                `aaagcaGTTACTGAGCGACCCCGATGAACTCGCTAAGGTCGATGAGGGCCTCTCGATGGTAGAGCGTATCATCATGAACTACATTGAGTCTAACAGTTACCGCGTGACTCTCTTTGAGGCTCTCAAACAGTTAGTCGTAGCTGGTAACGTCCTGCTGTACCTACCGGAACCGGAAGGGTCAAACTATAATCCCATGAAGCTGTACCGATTGTCTTCTTATGTGGTCCAACGAGACGCATTCGGCAACGTTCTGCAAATGGTGACTCGTGACCAGATAGCTTTTGGTTCTCTCCCTGAGGAc                       <  1:644643/301‑1 (MQ=255)`  
                                                                                                                                                                                                                                                                                   `gcaGTTACTGAGCGACCCCGATGAACTCGCTAAGGTCGATGAGGGCCTCTCGATGGTAGAGCGTATCATCATGAACTACCTTGAGTCTAACAGTTACCGCTTGACTCTCTTTGAGGCTCTCAAACCGTTAGTCATAGCTGGTAACGTCCTGCTGTACCTACCCGAACCCGAAGGGTCAAACTATAATCCCATGAAGCTGTACCGCTTGTTTTCTTATTTGTTCCAACGAGACGCATTCGGCAACGTTCTTCAAATGGTGACTCGTGACCAGATAGCTTTTGGTGCTCTCCCTGAGGAcctt                    >  1:3994/1‑298 (MQ=255)`  
                                                                                                                                                                                                                                                                                   `gcaGTTACTGAGCGACCCCGATGAACTCGCTAAGGTCGATGAGGGCCTCTCGATGGTAGAGCGTATCATCATGAACTACATTGAGTCTAACAGTTACCGCGTGACTCTCTTTGAGGCTCTCAAACAGTTAGTCGTAGCTGGTAACGTCCTGCTGTACCTACCGGAACCGGAAGGGTCAAACTATAATCCCATGAAGCTGTACCGATTGTCTTCTTATGTGGTACAACGAGACGCATTCGGCAACGTTCTGCAAATGGTGACTCGTGACCAGATAGATTTTGGTGCTCTCCCTGAGGACATc                    >  1:815221/1‑301 (MQ=255)`  
                                                                                                                                                                                                                                                                                      `gTTCCTGAGCGACCCCGATGAACTCGCTAAGGTCGATGAGGACCTCTCGATGGTAGAGCGTATCATCATGAACTACATTGAGTCTAACAGTTACCGCGTGACTCTCTTTGAGGCTCTCAAACAGTTAGTCGTAGCTGGTAACGTCCTGCTGTACCTACCGGAACCGGAAGGGTCAAACTATAATCCCATGAAGCTGTACCGATTGTCTTCTTATGTGGTCCAACGAGACGCATTCGGCAACGTTCTGCAAATGGTGACTCGTGACCAGATAGCTTTTGGTGCTCTCCCTGAGGACATCCGt                 >  1:268997/1‑301 (MQ=255)`  
                                                                                                                                                                                                                                                                                        `tACTGAGCGACCCCGATGAACTCGCTAAGGTCGATGAGGGCCGCTCGATGGGAGAGCGTATCATCATGAACTACATTGAGTCTAACAGTTACCGCGTGACTCTCTTTGAGGCTCTCAAACAGTTAGTCGTAGCTGGTAACGTCCTGCTGTACCTACCGGAACCGGAAGGGTCAAACTAGAATCCCATGAAGCTGTACCGATTGTCTTCTTATGTGGTCCAACGAGACGCATTCGGCAACGTTCTGCAAATGGTGACTCGTGACCAGATAGCTTTTGGTGCTCTCCCTGAGGACATCCGTaa               <  1:176270/301‑1 (MQ=255)`  
                                                                                                                                                                                                                                                                                          `cTGAGCGACCCCGATGAACTCGCTAAGGTCGATGAGGGCCTCTCGATGGTAGAGCGTATCATCATGAACTACATTGAGTCTAACAGTTACCGCGTGACTCTCTTTGAGGCTCTCAAACAGTTAGTCGTAGCTGGTAACGTCCTGCTGTACCTACCGGAACCGGAAGGGTCAAACTATAATCCCATGAAGCTGTACCGATTGTCTTCTTATGTGGTCCAACGAGACGCATTCGGCAACGTTCTGCAAATGGTGACTCGTGACCAGATAGCTTTTGGTGCTCTCCCTGAGGACATCCGTAAgg             <  1:13678/301‑1 (MQ=255)`  
                                                                                                                                                                                                                                                                                           `tGAGCGACCCCGATGAACTCGCTAAGGTCGATGAGGGCCTCTCGATGGTAGAGCGTATCATCATGAACTACATTGAGTCTAACAGTTACCGCGTGACTCTCTTTGAGGCTCTCAAACAGTTAGTCGTAGCTGGTAACGTCCTGCTGTACCTACCGGAACCGGAAGGGTCAAACTATAATCCCATGAAGCTGTACCGATTGTCTTCTTATGTGGTCCAACGAGACGCATTCTGCAACGTTCTGCAAATGGTGACTCGTGACCAGATAGCTTTTGGTGCTCTCCCTGAGGACATCCGTAAGGc            <  1:56089/301‑1 (MQ=255)`  
                                                                                                                                                                                                                                                                                           `tGAGCGACCCCGATGAACTCGCTAAGGTCGATGAGGGCCTCTCGATGGTAGAGCGTATCATCATGAACTACATTGAGTCTAACAGTTACCGCGTGACTCTCTTTGAGGCTCTCAAACAGTTAGTCGTAGCTGGTAACGTCCTGCTGTACCTACCGGAACAGGAAGGGTCAAACTATAATCCCATGAAGCTGTACCGATTGTCTTCTTATGTGGTCCAACGAGACGCATTCGGCAACGTTCTGCAAATGGTGACTCGTGACCAGATAGCTTTTGGTGCTCTCCCTGAGGACATCCGTAAGGc            <  1:566827/301‑1 (MQ=255)`  
                                                                                                                                                                                                                                                                                            `gAGCGACCCCGATGAACTCGCTAAGGTCGATGAGGGCCTCTCGATGGTAGAGCGTATCATCATGAACTACATTGAGTCTAACAGTTACCGCGTGACTCTCTTTGAGGCTCTCATACAGTTAGTCGTAGCTGGTAACGTCCTGCTGTACCTACCGGAACCGGAGGGGTCAAACTATAATCCCATGAAGCTGTAACGATTGTCTTCTTATGTGGTCCAACGAGACGCATTCGGCAACGTTCTGCAAATGGTGAAGCGTGACCAGATAGCATTTGGTGCTCTCCATGAGGACATCAGTAAGGcg           >  1:483748/1‑300 (MQ=255)`  
                                                                                                                                                                                                                                                                                                 `aCCCCGATGAACTCGCTAAGGTCGATGAGGGCCTCTCGATGGTAGAGCGTATCATCATGAACTACATTGAGTCTAACAGTTACCGCGTGACTCTCTTTGAGGCTCTCAAACAGTTAGTCGTAGCTGGTAACGTCCTGCTGTACCTACCGGAACCGGAAGGGTCAAACTATAATCCCATGAAGCTGTACCGATTGTCTTCTTATGTGGTCCAACGAGACGCATTCGGCAACGTTCTGCAAATGGTGACTCGTGACCAGATAGCTTTTGGTGCTCTCCCTGAGGACATCCGTAAGGCTGTAGa      <  1:86325/301‑1 (MQ=255)`  
                                                                                                                                                                                                                                                                                                 `aCCCCGATGAACTCGCTAAGGTCGATGAGGGCCTCTCGATGGTAGAGAGTATCATCATGAACTACATTGGGTCTAACAGTTAGCGCGTGACTCTCTTTGAGGCTCTCAAACAGTTAGTCGTAGCTGGTAACGTCCTGCTGTACCTACCGGAACCGGAAGGGTCAAACTATAATCCCATGAAGCTGTACCGATTGTCTTCTTATGTGGTCCAACGAGACGCATTCGGCAACGTTCTGCAAATGGTGACTCGTGACCAGATAGCTTTTGGTGCTCTCCCTGAGGACATCCGTAAGGCTGTAGa      <  1:703139/301‑1 (MQ=255)`  
                                                                                                                                                                                                                                                                                                     `cGATGAACTCGCTAAGGTCGATGAGGGCCTCTCGATGGTAGAGCGTATCATCATGAACTACATTGAGTCTAACAGTTACCGCGTGACTCTCTTTGAGGCTCTCAAACAGTTAGTCGTAGCTGGTAACGTCCTGCTGTACCTACCGGAACCGGAATGGTCAAACTATAATCCCATGAAGCTGTACCGATTGTCTTCTTATGTGGTCCAACGAGACGCATTCGGCAACGTTCTGCAAATGGTGACTCGTGACCAGATAGCTTTTGGTGCTCTcccctgtctcttatacacatctccgagcccc  >  1:492283/1‑273 (MQ=255)`  

                                                                                                                                                                                                                                                                                                          `|`                                                                                                                                                                                                                                                                                                         
`GAGAAACGAACAGGACTTGCGGAGGATGGCGCAAAGTCTGTCTATGAGCGTTTAAAGAACGACCGTGCTCCCTATGAGACACGCGCTCAGAATTGCGCTCAATATACCATCCCATCATTGTTCCCTAAGGACTCCGATAACGCCTCTACAGATTATCAAACTCCGTGGCAAGCCGTGGGCGCTCGTGGTCTGAACAATCTAGCCTCTAAGCTCATGCTGGCTCTATTCCCTATGCAGACTTGGATGCGACTTACTATATCTGAATATGAAGCAAAGCAGTTACTGAGCGACCCCGATGGACTCGCTAAGGTCGATGAGGGCCTCTCGATGGTAGAGCGTATCATCATGAACTACATTGAGTCTAACAGTTACCGCGTGACTCTCTTTGAGGCTCTCAAACAGTTAGTCGTAGCTGGTAACGTCCTGCTGTACCTACCGGAACCGGAAGGGTCAAACTATAATCCCATGAAGCTGTACCGATTGTCTTCTTATGTGGTCCAACGAGACGCATTCGGCAACGTTCTGCAAATGGTGACTCGTGACCAGATAGCTTTTGGTGCTCTCCCTGAGGACATCCGTAAGGCTGTAGAAGGT  >  NC_001604.1/20246‑20839`

  

Alignment Legend

Aligned base mismatch/match (shaded by quality score): `ATCG/ATCG` `< 3 ≤` `ATCG/ATCG` `< 15 ≤` `ATCG/ATCG` `< 27 ≤` `ATCG/ATCG` `< 37 ≤` `ATCG/ATCG` `< 38 ≤` `ATCG/ATCG`

Unaligned base: `atcg`    Masked matching base: `atcg`    Alignment gap: `‑`    Deleted base: `‑`
